# Transcriptome Analysis Reveals Different Responsive Patterns to Nitrogen Deficiency in Two Wheat Near-Isogenic Lines Contrasting for Nitrogen Use Efficiency

**DOI:** 10.3390/biology10111126

**Published:** 2021-11-03

**Authors:** Xinbo Zhang, Quan Ma, Fujian Li, Yonggang Ding, Yuan Yi, Min Zhu, Jinfeng Ding, Chunyan Li, Wenshan Guo, Xinkai Zhu

**Affiliations:** 1 Jiangsu Key Laboratory of Crop Genetics and Physiology/Jiangsu Key Laboratory of Crop Cultivation and Physiology, Agricultural College of Yangzhou University, Yangzhou 225009, China; zhxb202@126.com (X.Z.); mq_agriculture@163.com (Q.M.); fjli_agriculture@163.com (F.L.); 15949083474@163.com (Y.D.); minzhu@yzu.edu.cn (M.Z.); jfdin@yzu.edu.cn (J.D.); licy@yzu.edu.cn (C.L.); guows@yzu.edu.cn (W.G.); 2 Jiangsu Xuhuai Regional Institute of Agricultural Science, Xuzhou 221131, China; yvonneyi19890803@163.com; 3 Co-Innovation Center for Modern Production Technology of Grain Crops, Yangzhou University, Yangzhou 225009, China; 4 Joint International Research Laboratory of Agriculture and Agri-Product Safety, The Ministry of Education of China, Yangzhou University, Yangzhou 225009, China

**Keywords:** near-isogenic lines (NILs), nitrogen use efficiency (NUE), nitrogen deficiency, RNA sequencing (RNA-seq), qRT-PCR

## Abstract

**Simple Summary:**

Nitrogen (N) limitation is the key factor for wheat production worldwide. Therefore, the development of genotypes with improved nitrogen use efficiency (NUE) is a prerequisite for sustainable and productive agriculture. Exploring the molecular mechanisms of low N stress tolerance is significant for breeding wheat cultivars with high NUE. To clarify the underlying molecular mechanisms of enhanced resilience to low N in high-NUE wheat, we performed an RNA sequencing (RNA-seq) analysis. In the current research, two wheat near-isogenic lines (NILs) differing dramatically in NUE were used to measure gene expression differences under different N treatments. There was a dramatic difference between two wheat NILs in response to N deficiency at the transcriptional level, and the classification of identified candidate genes may provide new valuable insights into the resilience mechanism of wheat.

**Abstract:**

The development of crop cultivars with high nitrogen use efficiency (NUE) under low-N fertilizer inputs is imperative for sustainable agriculture. However, there has been little research on the molecular mechanisms underlying enhanced resilience to low N in high-NUE plants. The comparison of the transcriptional responses of genotypes contrasting for NUE will facilitate an understanding of the key molecular mechanism of wheat resilience to low-N stress. In the current study, the RNA sequencing (RNA-seq) technique was employed to investigate the genotypic difference in response to N deficiency between two wheat NILs (1Y, high-NUE, and 1W, low-NUE). In our research, high- and low-NUE wheat NILs showed different patterns of gene expression under N-deficient conditions, and these N-responsive genes were classified into two major classes, including “frontloaded genes” and “relatively upregulated genes”. In total, 103 and 45 genes were identified as frontloaded genes in high-NUE and low-NUE wheat, respectively. In summary, our study might provide potential directions for further understanding the molecular mechanism of high-NUE genotypes adapting to low-N stress.

## 1. Introduction

Wheat (*Triticum aestivum* L.) is one of the important major cereal crops all over the world and plays a crucial role in agricultural productivity [1]. Nitrogen (N) is the most essential macronutrient required for the growth and development of plants [2]. N limitation has a serious impact on the morphology, limiting the growth and reducing biomass in wheat [3]. In the past several decades, N fertilizer has been put into farmland in large amounts to meet increasing food demands [4]. The current increase in production cost is largely due to the excessive use of N fertilizer in agriculture. However, further yield increases through an additional N fertilizer supply are very limited. In addition, nitrogen use efficiency (NUE) is very low in wheat, as only less than half of the N fertilizer applied could be used by crops [5], and the remaining N causes serious environmental pollution and resource waste [6,7]. Excessive fertilizer application has become a major problem in crop production [8], and there is an urgent need to reduce the rate of N application without compromising crop yields. Notably, an appropriate reduction in nutrient supply does not necessarily result in a dramatic decrease in yield and may stimulate more efficient use of this application [9].

NUE is a complex trait that is controlled genetically [10], and it is usually considered to be the amount of dry biomass or the yield of grain produced per unit of the applied N fertilizer [11]. Agriculturally, crops take up N from the soil primarily in two forms: nitrate and ammonium. Meanwhile, nitrate also acts as signal molecules, inducing changes to genes involved in N assimilation and transport, like nitrate transporters (NRT), glutamine synthetase (GS), and glutamate synthase (GOGAT) [12,13]. To make full use of N fertilizer more efficiently and economically, many agricultural strategies have been tried to reduce the amount of N fertilizer usage while maintaining normal levels of crop yield [14]. Additionally, plant genetic information is also an important approach to improve NUE [15]. For instance, Shah et al. [16] reported that the NRT2.1 expression level in the N-efficient genotypes was higher than that in the inefficient ones in barley under low-N stress. Likewise, Wei et al. [17] found that overexpression of TaGS2 may improve nitrogen assimilation under N-deficit conditions. Therefore, it is of great importance to explore the potential candidate genes for high-NUE cultivar breeding.

It has been well documented that the understanding of the molecular regulatory mechanism of plants in response to N limitation is an effective approach to improve NUE [18,19]. In recent years, RNA sequencing (RNA-seq) has been extensively used to identify N-responsive genes and enhance plants’ ability to use N more efficiently [20,21]. This technique has been employed broadly in various crop species. For instance, Liu et al. [22] employed RNA-seq to analyze transcriptomic changes in wheat under N-deficient conditions and identified new candidate genes, which can directly promote wheat growth. In another study, Wei et al. [23] performed a transcriptome analysis of the leaf and identified key genes related to the metabolism of carbon and nitrogen in rice seedlings under different N treatments. Similarly, Schlüter et al. [24] revealed that N starvation resulted in the downregulation of genes responsible for nitrate reduction and amino acid assimilation in maize by using transcriptome analysis. Most research efforts have mainly concentrated on understanding the regulation of N-responsive gene expression in a single genotype under low-N stress. One limitation in these studies is that N stress and genotypes were not analyzed simultaneously. In this situation, a comprehensive transcriptome analysis of genotypes with contrasting NUE is particularly meaningful to narrow down the number of candidate genes for improving the NUE of wheat plants.

The development of near-isogenic lines (NILs) reduces genetic background noise in transcriptome studies. In the current study, we employed RNA-seq to investigate the difference in the gene expression profiles between two wheat NILs with contrasting NUE (1Y, high-NUE, and 1W, low-NUE) under low-nitrogen (no N supply) and normal conditions (1.6 g N pot^−1^). The main objectives of this study were to explore the gene expression patterns in two wheat NILs in response to N deficiency, and the functional categorization of DEGs was performed to reveal the key regulatory mechanisms of high-NUE wheat genotypes resisting N deficiency. 

## 2. Materials and Methods

### 2.1. Pot Experiment and Sampling

The pot experiment was carried out at the experimental station of Yangzhou University in China (32°23′ N, 119°25′ E) during the 2019–2020 growing seasons. A set of wheat NILs was bred via crossing and back-crossing with P7001 and P216. The NUE of 1W and 1Y was 33.41 and 49.33%, respectively, according to the previous study [25]. In this study, two wheat NILs (1W and 1Y) were selected as the experimental materials. The soil was obtained from the top 20 cm horizon at the experimental site, and the pH of the test soil was 7.26. The nutrient substances in the soil were 16.4 g kg^−1^ organic C, 117.4 mg kg^−1^ available N, 54.8 mg kg^−1^ available P, and 124.3 mg kg^−1^ available K. Twelve kilograms of the soil were loaded into a plastic pot (top diameter 26 cm, height 26.5 cm). The soil was air-dried, sieved through 8 mm, and mixed with fertilizer before loading into the pots.

Two N fertilizer treatments were designed, and each treatment consisted of 10 replicate pots. Nitrogen treatments consisted of two contrasting levels (0 and 300 kg ha^−1^, equivalent amount of 0 and 1.6 g pot^−1^), which were referred to as N0 and N1. We selected urea, calcium-magnesium phosphate, and potassium chloride as the mineral N, P, and K fertilizers, respectively. Urea was applied in three splits: 50% as basal fertilizer, 10% at the four-leaf stage, and the remaining 40% at the jointing stage. Meanwhile, each pot received an application of P and K at the rate of 150 kg ha^−1^ (equivalent amount of 0.8 g pot^−1^) as basal fertilizers in all treatments, respectively. Twelve uniform seeds of each genotype were sown in each pot on 31 October 2019, and eight plants were kept at the stage of three-leaf. Irrigation, weeding, and insecticide were used according to the standard agronomic practices for all pots.

At the anthesis stage, five pots of plant samples from each treatment were harvested and the different tissues were preserved after being separated into the stem, leaf, and spike. Each part was oven-dried for biomass and N content measurements. The Kjeldahl method was used for total N content analysis [26]. Flag leaves were collected from each treatment for the measurement of nitrate reductase (NR), glutamine synthetase (GS), and glutamate synthase (GOGAT) activity, and each sample contained three biological replicates. The activities of NR, GS, and GOGAT were assayed at a wavelength of 340, 540, and 340 nm, respectively, using the corresponding assay kits (Cominbio Biotech, Suzhou, China) according to the manufacturer’s protocol. Meanwhile, a total of four genotype-condition combinations, namely N0_1W, N0_1Y, N1_1W, and N1_1Y, were used for RNA extraction, respectively. The collected leaf tissues from each treatment were immediately frozen in liquid nitrogen with silver paper and stored in −80 °C conditions for the following analysis. 

### 2.2. RNA Extraction, Library Construction, and Transcript Profiling

Total RNA was extracted from the wheat leaves using the RNA simple Total RNA Kit (Tiangen Biotech, Beijing, China) according to the protocol provided by the manufacturer. The purity and concentration of RNA were verified by a nanodrop spectrophotometer (Thermo Scientific, Waltham, MA, USA) and Agilent Bioanalyzer 2100 system (Agilent Technologies, Santa Clara, CA, USA), respectively. The RNA-sequencing libraries were constructed using the TruSeqTM RNA Sample Preparation Kit (Illumina, San Diego, CA, USA) following the manufacturer’s recommendations. Finally, a total of 12 cDNA libraries were sequenced, and 150 bp paired-end sequencing reads were generated via the Illumina sequencing platform. For further analysis, the clean reads were obtained by removing low-quality reads, reads containing adapters, and reads containing ploy-N sequences from raw data. Hisat2 (version 2.1.0) software [27] was then used to align the obtained clean reads of each sample to the Chinese Spring wheat reference genome.

The transcription levels of each gene were normalized using the fragments per kilobase of transcript per million mapped reads (FPKM) method [28]. Differential gene expression analysis was performed in the R environment using the package DESeq2 (1.16.1) [29], and genes with an adjusted *p*-value of less than 0.05 and an absolute log2-ratio larger than 1 were considered to be statistically differentially expressed. The functions of the identified genes were predicted via BLASTx searches against the NR, NT, Uniprot, and Pfam databases. Gene ontology (GO) [30] and Kyoto Encyclopedia of Genes and Genomes (KEGG) [31] analysis were performed to identify significant enrichment in the GO terms and metabolic pathways of differentially expressed genes (DEGs) at *p*-value <0.05, respectively. The GO distribution associated with DEGs was classified into three categories, including biological process (BP), cellular component (CC), and molecular function (MF) [32].

### 2.3. Confirmation of RNA-Seq Data by qRT-PCR Analysis

To examine the gene expression profiles obtained from RNA-seq analysis, we randomly selected 12 DEGs for qRT-PCR validation. All primers listed in Table S1 were designed using NCBI Primer Blast. Total RNA from each sample was extracted as described above, and one microgram of total RNA was reverse transcribed into first-strand cDNA to be used for qPCR. Gene expression analysis was performed using the ChamQ Universal SYBR ^®^ qPCR Master Mix (Vazyme Biotech, Nanjing, China) and CFX96 Real-Time PCR Detection System (Bio-Rad, Hercules, CA, USA). The PCR procedure was performed at 95 °C for 30 s, 40 cycles of 95 °C for 10 s, and 60 °C for 30 s, followed by melt curve analysis (65–95 °C, with 0.5 °C increments every 5 s). Each gene was repeated three times per sample to ensure statistical reliability, and the relative expression levels were processed using the 2^−ΔΔCT^ approach with GAPDH as the reference [33]. Verification of the potential key DEGs related to NUE of wheat was performed as described above, and the sequence of primers are listed in Supplementary Materials Table S1.

### 2.4. Statistical Analysis

Data analysis was performed with SPSS 19.0 software (SPSS Inc., Chicago, IL, USA), and graphs were generated using Sigma Plot 10.0 software (Systat Software, Inc., San Jose, CA, USA). For statistical analysis, differences were analyzed at the 0.05 probability level according to the least significant difference (LSD) test. Data presented are means ± standard deviation (SD). 

## 3. Results

### 3.1. Influences of N Level on the Growth Parameters and Enzyme Activities of Wheat NILs

**Figure 1:**
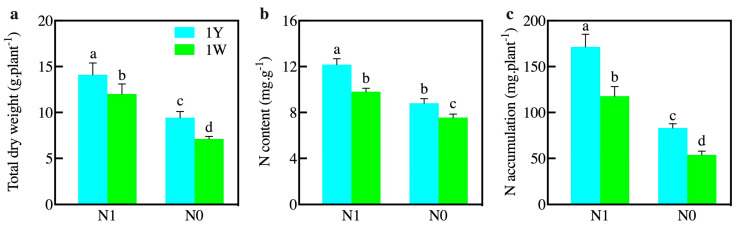
Plant growth performance of wheat NILs at the anthesis stage under different nitrogen treatments. (**a**) Total dry weight; (**b**) N content; (**c**) N accumulation; 1Y and 1W represent the high-NUE and low-NUE wheat, respectively; N1 and N0 represent the normal nitrogen and low nitrogen fertilizer application level, respectively. The results shown are the means +/− SD, different lowercase letters denote a statistically significant difference (*p* < 0.05).

One set of wheat NILs was used in the study, and 1Y and 1W were considered as high-NUE and low-NUE, respectively [25]. To determine the characteristics of 1Y and 1W under different N treatments, the growth performance was measured at the stage of anthesis. 1Y had a higher total dry weight and greater N accumulation in the leaf tissues in comparison with 1W under normal N conditions (N1). Meanwhile, the N content was significantly higher in 1Y than that in 1W (Figure 1). Both wheat NILs exhibited a significant decrease in total dry weight and N accumulation after the N-deficit stress treatment (N0). These results indicated that N deficiency restrained the growth of wheat despite the NUE performance. However, the total dry weight and N accumulation in 1Y was 1.32 and 1.54 times significantly more than 1W under N-deficit stress, respectively. It is noteworthy that the N content of 1Y was always higher than 1W, regardless of normal-N or low-N conditions (Figure 1). 

In the study of the specific activities of the three key N-assimilating enzymes in leaves, there were significant genotype differences between the two wheat NILs under normal N conditions. The activities of the enzymes were higher in 1Y than 1W (Figure S1). Furthermore, in comparison with normal N conditions, N deficiency had a direct negative effect on the nitrate assimilatory enzymes. In this study, NR activity was found to be reduced in the leaves of both wheat NILs under N-deficit conditions. The activity of GOGAT decreased under N deficiency in 1W, whereas no significant difference was shown in 1Y (Figure S1).

### 3.2. Analysis of Transcriptome Sequencing Data

To gain a better insight into the molecular mechanism of the two wheat NILs’ response to low-N stress, transcriptome sequencing was used to study the gene expression under different nitrogen treatments. Approximately 41.40 to 46.72 million raw reads were generated (Table 1). The average Q30 (%) ratio of the four groups was more than 93.84%, which met the requirements for further analysis. After quality control and data filtering, 42.36 and 46.45 million clean reads were obtained from N0_1W and N0_1Y samples, while N1_1W and N1_1Y samples engendered 41.14 and 44.76 million clean reads. The proportion of clean reads in the four groups ranged from 99.39% to 99.45%. From the comparison results, approximately 39.08 to 44.07 million clean reads were mapped to the wheat “Chinese Spring” reference genome (total mapped) in the four groups, and the percentage of total mapped was not less than 94.87%. Among the mapped reads, the uniquely mapped reads accounted for 94.15–94.76%. In summary, our data proved to be useful for the subsequent data analysis.

### 3.3. Identification and Validation of DEGs

**Figure 2:**
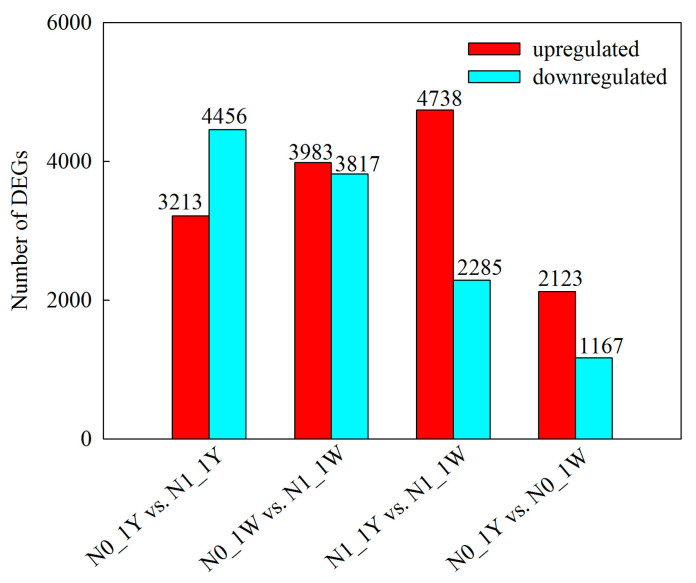
Number of DEGs in pairwise comparisons. The red and cyan colors of the graph represent the genes that are up- or downregulated, respectively.

Of the screened transcriptome data, 7669 and 7800 genes were significantly differentially expressed between N0_1Y and N1_1Y and between N0_1W and N1_1W, respectively. An additional 7023 DEGs were detected in the N1_1Y vs. N1_1W comparison, whereas 3290 DEGs were found in the N0_1Y vs. N0_1W comparison (Figure 2, Table S2). From these results, the largest number of DEGs was found between N0_1W and N1_1W among the four comparisons. In general, the number of upregulated DEGs was 1.04- to 2.07-fold higher than that of downregulated DEGs except for the N0_1Y vs. N1_1Y comparison (Figure 2).

**Figure 3:**
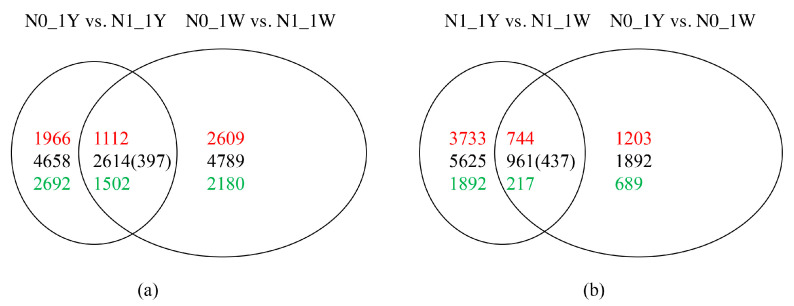
Venn diagram of the DEGs derived from different pairwise comparisons. The differentiations were compared between nitrogen treatments in each wheat NIL (**a**), or between wheat NILs under each nitrogen treatment (**b**). The overlapping section of the Venn diagram indicates common DEGs between the compared combinations. The black color represents the total numbers, and black numbers in parentheses represent genes with inconsistent expression trends. Color gradient red to green indicates the up- to downregulated genes, respectively.

Furthermore, Venn diagram analysis was carried out to identify the number of common and unique DEGs in the comparisons of the two N treatments. Through Venn’s analysis of the results, we found that 3011 of the 7800 DEGs between N0_1W and N1_1W were also among the genes differentially expressed between N0_1Y and N1_1Y. Meanwhile, 4658 genes were identified in the N0_1Y vs. N1_1Y comparison and 4789 genes in the N0_1W vs. N1_1W comparison (Figure 3a). Moreover, 1398 common DEGs were detected in both the N0_1Y vs. N0_1W and N1_1Y vs. N1_1W comparison. It was also observed that the unique DEGs in the normal conditions were nearly 2.97-fold higher than that in the N-deficit conditions (Figure 3b). Thus, we may assume that increasing the amount of N fertilizer application affected the genes’ expression more specifically. To validate the accuracy of the RNA-seq data, 12 DEGs were randomly chosen and checked using qRT-PCR. The results from the qRT-PCR analysis were generally in agreement with the RNA-seq trend, proving the reliability of the RNA-seq results (Figure S2).

### 3.4. Functional Characterization of the DEGs by GO and KEGG Pathway Analysis

To better explore the potential functions of all the DEGs, GO annotation and KEGG pathway analysis was performed. DEGs were classified into three main GO categories (biological process, cellular component, molecular function), and the distribution of the functional category had similar patterns among the different comparisons (Figure S3). In the category of BP, cellular process (GO: 0009987) was the most dominant group, followed by metabolic process (GO: 0008152). Within the CC group, the terms with the highest number of genes were cell part (GO: 0044464) and organelle (GO: 0043226). According to the MF category, most DEGs were involved in binding (GO: 0005488) and catalytic activity (GO: 0003824) (Figure S3).

The enriched GO terms showed that “photosynthesis, light-harvesting” (GO: 0009765), “glutathione metabolic process” (GO: 0006749), “photosystem I” (GO: 0009522), and “glutathione transferase activity” (GO: 0004364) were the most significantly enriched categories in the N0_1Y vs. N1_1Y comparison. The enriched GO terms in the N0_1W vs. N1_1W comparison were “organic acid transport” (GO: 0015849), “carboxylic acid transport” (GO: 0046942), “integral component of membrane” (GO: 0016021), and “transmembrane transporter activity” (GO: 0022857). Under normal N conditions, the GO terms significantly enriched in the N1_1Y vs. N1_1W comparison included “regulation of jasmonic acid-mediated signaling pathway” (GO: 2000022), “response to nitrogen compound” (GO: 1901698), “intrinsic component of membrane” (GO: 0031224), and “DNA-binding transcription factor activity” (GO: 0003700). Under N-deficient conditions, “glutathione metabolic process” (GO: 0006749), “integral component of membrane” (GO: 0016021), and “DNA-binding transcription factor activity” (GO: 0003700) were significantly enriched in the N0_1Y vs. N0_1W comparison (Table S3).

We further performed the enrichment analysis for DEGs according to the KEGG database. KEGG pathway enrichment analysis revealed that “MAPK signaling pathway”, “glutathione metabolism”, and “photosynthesis-antenna proteins” were significantly enriched in the N0_1Y vs. N1_1Y comparison (Figure S4a). “Glutathione metabolism”, “MAPK signaling pathway”, and “alpha-linolenic acid metabolism” were most highly enriched in the N0_1W vs. N1_1W comparison (Figure S4b). Meanwhile, “glutathione metabolism”, “MAPK signaling pathway”, and “alpha-linolenic acid metabolism” were also the most represented pathways in the N1_1Y vs. N1_1W comparison (Figure S4c). “Glutathione metabolism”, “plant hormone signal transduction”, and “MAPK signaling pathway” were detected in the N0_1Y vs. N0_1W comparison (Figure S4d).

### 3.5. Responses of Genes Involved in Nitrogen Metabolism to N-Deficit Conditions

**Figure 4:**
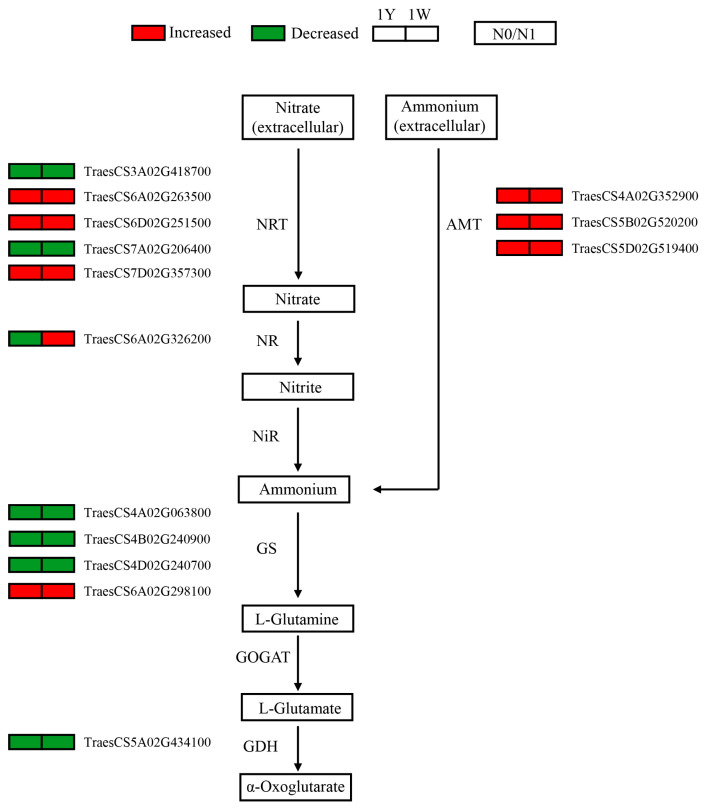
Nitrogen metabolism genes are responsive to N deficiency in each wheat NIL. The relative levels of these differential expression genes are indicated by red and green. Red boxes represent upregulated and green boxes denote downregulated. NRT, nitrate transporter; AMT, ammonium transporter; NR, nitrate reductase; Nir, ferredoxin-nitrite reductase; GS, glutamine synthetase; GOGAT, glutamate synthase; GDH, glutamate dehydrogenase.

Compared with normal N conditions, many genes associated with nitrogen absorption and ammonium assimilation were found to be differentially expressed under low-N stress. Here, five DEGs related to nitrate transporters (NRTs) were identified in the N0_1Y vs. N1_1Y and N0_1W vs. N1_1W comparison, of which, three DEGs showed increased gene expression levels and two DEGs showed decreased levels. Meanwhile, three genes involved in ammonium transporters (AMTs) were detected (Figure 4). Moreover, some DEGs encoding key enzymes in nitrate assimilation were found, including one nitrate reductase (NR), four glutamine synthetase (GS), and one glutamate dehydrogenase (GDH). It is noteworthy that most of the DEGs related to nitrate assimilation were decreased in the leaves of both 1Y and 1W (Figure 4).

### 3.6. Specific Responses of the High-NUE Wheat to N-Deficit Conditions

**Figure 5:**
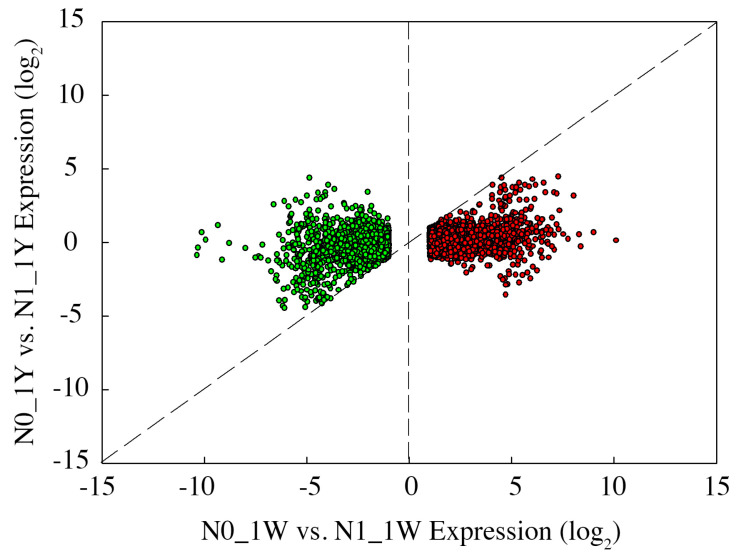
Scatter plot comparing the expression levels for the 4725 genes specifically expressed in the N0_1W vs. N1_1W comparison between N treatments in each wheat NIL. Each point represents an individual DEG, and the red and green colors of the graph represent the genes that are upregulated or downregulated.

In the current study, 1Y and 1W showed different transcriptome responses to N-deficit conditions. In 1W, 4789 unique genes were significantly responsive to N deficiency, including 2609 upregulated and 2180 downregulated genes (Figure 3a). There were 4764 genes in 1W (excluding 25 genes not expressed) that were not significantly differentially expressed in 1Y. These genes had no significant difference in 1Y due to the failure to meet the criteria of the absolute log_2_ (fold change) ≥1 or an adjusted *p*-value <0.05. Through analysis of these genes’ expression levels, we found that a total of 4725 genes (excluding 39 genes) in 1W were expressed at higher (2584 upregulated) or lower (2141 downregulated) levels than 1Y (Figure 5). Further, we tried to understand these 4725 genes’ expression patterns between 1Y and 1W under different N treatments.

**Figure 6:**
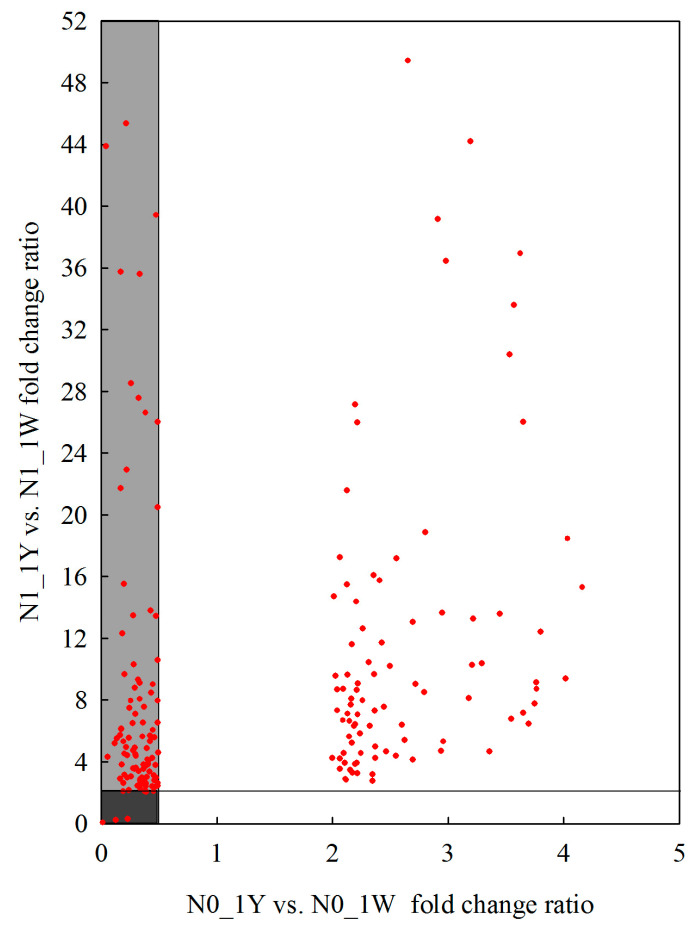
Scatter plot comparing the relative expression ratio for the 203 upregulated genes specifically expressed in the N0_1W vs. N1_1W comparison between wheat NILs under each N treatment. Each point represents an individual DEG, and the lighter section of the diagram shows the genes that are potentially frontloaded in expression.

**Figure 7:**
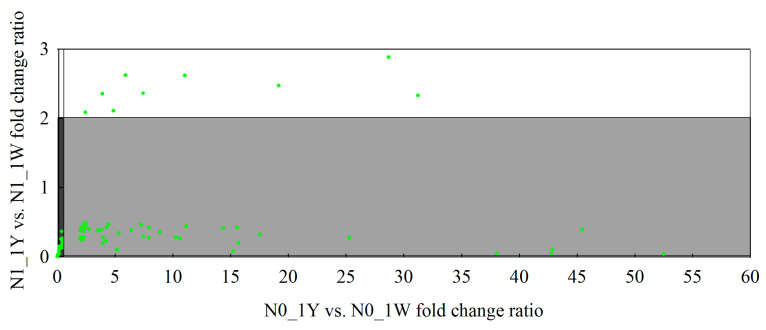
Scatter plot comparing the relative expression ratio for the 72 downregulated genes specifically expressed in the N0_1W vs. N1_1W comparison between wheat NILs under each N treatment. Each point represents an individual DEG, and the lighter section of the diagram shows the genes that are potentially relatively upregulated in expression.

In this study, we found that 203 of the 2584 upregulated genes were also among the genes differentially expressed in the N1_1Y vs. N1_1W and N0_1Y vs. N0_1W comparisons by using Venn’s analysis. Among them, 103 of the 203 genes in 1Y had higher expression in the N1_1Y vs. N1_1W (fold change ratio > 2) but lower expression in the N0_1Y vs. N0_1W (fold change ratio < 0.5) (Figure 6, Table S4). This relationship suggested that 103 DEGs showed higher gene expression levels in 1Y before the onset of N-deficiency stress, which might react less sensitively under N-deficit conditions. Among the 2141 downregulated DEGs in 1W, 72 genes were co-expressed in the N1_1Y vs. N1_1W and N0_1Y vs. N0_1W comparisons through Venn’s analysis. Furthermore, 43 of the 72 DEGs in 1Y had lower expression in the N1_1Y vs. N1_1W (fold change ratio < 0.5) but higher expression in the N0_1Y vs. N0_1W (fold change ratio > 2) (Figure 7, Table S4). The analysis indicated that 43 DEGs were expressed at higher levels in 1Y after the onset of N-deficiency stress.

### 3.7. Specific Responses of the Low-NUE Wheat to N-Deficit Conditions

In contrast, a total of 4658 (1966 upregulated and 2692 downregulated) specific DEGs were significantly responsive to N deficiency in 1Y (Figure 3a). There were 4595 genes in 1Y (excluding 63 genes not expressed) that were not significantly differentially expressed in 1W, of which 4534 genes (excluding 61 genes) had higher (1905 upregulated) or lower (2629 downregulated) expression in 1Y than in 1W (Figure S5). Further, we tried to understand these 4534 genes’ expression patterns between 1W and 1Y under different nitrogen treatments by using Venn’s analysis. In total, 95 of the 1905 upregulated genes were detected in the N1_1W vs. N1_1Y and N0_1W vs. N0_1Y comparisons. Among them, 45 of the 95 genes in 1W showed higher expression in the N1_1W vs. N1_1Y (fold change ratio > 2) but lower expression in the N0_1W vs. N0_1Y (fold change ratio < 0.5), respectively, indicating that these 45 DEGs in 1W were expressed at higher levels before responding to N-deficiency stress (Figure S6, Table S5). Among the 2629 downregulated DEGs in 1Y, there were 93 common DEGs in the N1_1W vs. N1_1Y and N0_1W vs. N0_1Y comparisons. Additionally, we found that 50 of the 93 DEGs in 1W showed lower expression in the N1_1W vs. N1_1Y (fold change ratio 0.5) but higher expression in the N0_1W vs. N0_1Y (fold change ratio > 2) (Figure S7, Table S5), suggesting that these 50 DEGs in 1W were expressed at higher levels after the onset of N-deficit conditions.

### 3.8. Classification of N-Responsive Genes in Wheat

In the present study, the genetic difference of the transcriptomic profiles of 1Y and 1W wheat NILs in response to N deficiency was analyzed, and we found that the expression patterns of these N-responsive genes were different between N treatments in each wheat NIL (Figure 3a). These N-responsive genes were classified into two major classes based on the differential expression levels: “frontloaded genes”, which displayed higher genes expression levels between the two wheat NILs under normal nitrogen conditions but exhibited a lower response to N-deficiency stress; and “relatively upregulated genes”, which exhibited relatively higher expression levels between the two wheat NILs under N deficiency but showed lower expression patterns under normal N conditions. The transcriptome analysis of these two types of genes could provide a molecular basis for understanding high NUE in wheat.

Based on the classification of N-responsive genes as described above, we identified 103 frontloaded genes and 43 relatively upregulated genes in 1Y (Table S4). Functional annotation showed that these genes could be further separated into 36 groups (Figure S8), including “single-organism process” (107 genes), “metabolic process” (102 genes), “cell part” (113 genes), “cell” (113 genes), and “binding” (96 genes). In contrast, we identified 45 frontloaded genes and 50 relatively upregulated genes in 1W (Table S5). These genes were classified into 35 functional groups (Figure S8) through GO analysis, including “metabolic process” (64 genes), “cell” (68 genes), “cell part” (68 genes), and “binding” (61 genes). In general, we identified 146 genes in 1Y and 95 genes in 1W. The frontloaded genes in 1Y were 2.28 times significantly higher than that of 1W. Meanwhile, there were no significant differences in the relatively upregulated genes between 1Y and 1W. A total of 12 potential key genes were randomly selected and further validated by qRT-PCR analysis, of which nine were frontloaded genes and three were relatively upregulated genes (Figure S9). Correlation analysis showed that the qRT-PCR expression patterns of the 12 genes were highly consistent with the RNA-seq analysis (*R*^2^ = 0.7876; Figure S10).

## 4. Discussion

Nitrogen (N) is a primary component in fertilizers for plant growth and development, and the use of N fertilizer has contributed to the increase in crop production [34]. As the technology of “nitrogen-saving and efficiency-increasing” has been popularized, a combination of low N supply and high yield should be applied for sustainable agriculture. Previous studies have shown that there were large differences in NUE among different genotypes in wheat [35], and understanding the mechanisms of plants’ response to N limitation is an efficient approach for improving NUE [18]. Recently, several transcriptome analyses have been carried out to characterize the gene expression responses to low-N stress in many crops, such as wheat and rice [36,37]. However, these studies focused on gene expression in a single genotype. Therefore, in our research, we considered N stress simultaneously with genotypes. In the present study, the differences in total dry weight and N accumulation were significant between two wheat NILs under different N treatments (Figure 1). These results suggested that high-NUE wheat had a distinctively higher level of resilience to nitrogen stress. Additionally, transcriptional alterations in two wheat NILs were investigated to better understand the molecular mechanisms in response to different N treatments. Moreover, we identified the candidate DEGs that enhance resilience in high-NUE wheat under N-deficient conditions and categorized these genes into two major classes.

### 4.1. Nitrogen Metabolism Genes Responsive to N-Deficient Conditions

As we know, extracellular inorganic nitrogen enters into the cell through the nitrate transporters (NRTs) or ammonium transporters (AMTs) system, and the majority of nitrate is assimilated in plant leaves [12]. Nitrate needs to be reduced to nitrite by nitrate reductase (NR) and catalyzes nitrite to ammonium by nitrite reductase (NiR). The ammonium is integrated into the structure of amino acids via the GS/GOGAT cycle, which is carried out by glutamine synthetase (GS) and glutamate synthase (GOGAT) in plants [13]. In most cases, N deficiency enhanced the expression level of transports for nitrate and ammonium [38]. Similarly, our results showed that most of these genes encoding NRTs and AMTs had higher expression under the N-deficient relative to the normal conditions (Figure 4). Previous studies reported that N assimilation required energy and carbon skeletons produced by the leaves during respiration [39], and N starvation caused a dramatic decrease both in N-assimilating enzyme activity and related gene expression levels [40]. In the current study, many kinds of genes related to the key enzymes were downregulated, suggesting a reduction in the N assimilation capacity (Figure 4). This countermeasure might be useful to maintain the efficiency of the energy supply.

### 4.2. Some Potential Genes for High-NUE Wheat Response to N-Deficient Conditions

In this study, the identified frontloaded genes in high-NUE wheat were 2.28 times significantly higher than that of low-NUE wheat, and there were no significant differences between the relatively upregulated genes of the two wheat NILs. Therefore, we mainly discuss the possible mechanisms of the frontloaded genes in the following paragraphs.

Among the frontloaded genes identified, some might play a key role in responding to N deficiency, such as amino acid transporters, antioxidant enzymes, protein kinases, and transcription factors. As we know, amino acid transports (AATs) perform the critical function of transporting amino acids through the cell membrane for plant growth [41]. In the current study, one gene encoding AATs was detected. Thus, there is considerable potential to manipulate the expression of amino acid transporters and further improve NUE in wheat [42].

In general, abiotic stresses cause rapid accumulation of reactive oxygen species (ROS) in many cell components, such as lipids, proteins, and nucleic acids, which leads to tissue damage in plants [43]. To cope with the harmful oxidative stress, plant cells possess an efficient enzymatic antioxidant defense system to regulate the level of ROS [44]. Superoxide dismutase (SOD), peroxidases (PODs), and catalases (CATs) are important antioxidant enzymes, which exhibit effective ROS scavenging ability [45]. Wang et al. [3] reported that the genes encoding POD were expressed at high levels in wheat under low-N stress. Furthermore, cytochrome P450s (CYPs) are involved in various physiological processes through biosynthesis and detoxification pathways in plants [46]. Quan et al. [38] found nine CYP genes that showed higher expression in LN-tolerant wild barley under N deficiency. Currently, in the present work, two and six DEGs encoding POD and CYPs were found in high-NUE wheat, respectively (Table S4), indicating a higher capacity of antioxidant defense.

Protein kinases (PKs) regulate transcription [47] and play a vital role in the adaptation of plants to abiotic stresses [48]. For example, TaMPK14 is an important gene involved in the modulation of wheat tolerance to low-N stress, realizing this function through the regulation of NRT genes [49]. Previous research indicated that receptor-like protein kinases (RLKs) are involved in signal transduction pathways associated with abiotic stress stimuli in plants [50]. Besides, overexpression of the genes encoding CBL-interacting protein kinase (CIPK) enables rice to exhibit a higher NO_3_^−^ uptake capacity under low-N stress [51]. In the present study, different groups of PK genes were identified, which mainly included MAPK, RLK, and CIPK families (Table S4). Notably, we observed that the genes related to the MAPK family were highly abundant among the PK families. Thus, we may hypothesize that the genes associated with PK may contribute to its high NUE in wheat.

A previous study showed that some transcription factors (TFs) participate in the control of transcriptional regulation of several genes associated with nitrogen metabolism in plants [52]. Remarkably, it has been reported that 170 genes encoding TFs have been identified in wheat under N deficiency [47]. Heerah et al. [53] revealed that WRKY1 regulates the expression of various nitrogen-related genes, including NRT2.1 and AMT1.1 genes, in Arabidopsis. Wang et al. [54] reported that the expression of TIFY10c was promoted under N deficiency in wheat. Similarly, overexpression of TabHLH1 enhances the expression of NRT2.2 and several genes involved in the antioxidant enzyme under low-N stress in wheat [55]. In this study, among these frontloaded genes, TF families, such as WRKY, TIFY, bHLH, and ERF, were identified that might be responsible for the transcriptional activation of N-responsive genes related to N stress. Altogether, these identified TF genes might cast a light on the regulation of wheat responses to N deficiency.

## 5. Conclusions

Identification of DEGs in plants would be helpful to uncover the underlying molecular mechanisms under N-deficiency stress. Our results showed that there was a significant difference in the transcriptomic response to low-N conditions between two wheat NILs with contrasting NUE. The N-responsive genes were classified into two major classes according to their expression patterns, which developed the potential molecular mechanism of the wheat response to low-N stress. The current study identified 103 frontloaded genes in high-NUE wheat and 45 genes in low-NUE wheat, and we deduced that the significantly increased frontloaded genes at the molecular level may explain the high NUE in wheat. In addition, some new potential candidate genes could be useful for improving the NUE of wheat.

## Figures and Tables

**Table 1:** RNA sequencing reads and mapped to the wheat genome.

Samples	Raw Reads	Q30 (%)	Clean Reads	Clean Reads %	Total Mapped	Uniquely Mapped
N0_1W	42,598,731	94.81	42,364,637	99.45	40,389,624 (95.34%)	38,025,789 (94.15%)
N0_1Y	46,724,559	94.49	46,447,429	99.41	44,065,654 (94.87%)	41,507,838 (94.20%)
NI_1W	41,396,203	93.84	41,144,746	99.39	39,078,506 (94.98%)	37,029,109 (94.76%)
NI_1Y	45,007,941	94.62	44,758,766	99.44	42,670,214 (95.33%)	40,297,380 (94.44%)

Sample: sample’s name; N0_1W: 1W under N-deficient conditions; N0_1Y: 1Y under N-deficient conditions; N1_1W: 1W under normal nitrogen conditions; N1_1Y: 1Y under normal nitrogen conditions; Raw Reads: total number of sequencing reads; Q30 (%): the percentage of bases with a base recognition accuracy of 99% or more; Clean Reads: the reads of filtered raw reads; Clean Reads (%): clean reads/raw reads; Total Mapped: total number of sequences of the wheat reference genome on the alignment, the percentage is total mapped/clean reads; Uniquely Mapped: reads mapped to unique genomic locations, the percentage is uniquely mapped/total mapped.

## Data Availability

Raw sequencing data can be accessed through the Gene Expression Omnibus with the accession number GSE179179.

## Supplementary Materials

The following are available online at https://www.mdpi.com/article/10.3390/biology10111126/s1, Figure S1: The activities of key enzymes related with N metabolism in the leaves of two wheat NILs under different N treatments; Figure S2: qRT-PCR validation of the RNA-seq data of the 12 randomly selected DEGs; Figure S3: Distribution of DEGs among the GO terms in the three main categories; Figure S4: Analysis of KEGG enrichment in four pairwise groups; Figure S5: Scatter plot comparing the expression levels for the 4534 genes specifically expressed in the N0_1Y vs. N1_1Y comparison between N treatments in each wheat NIL; Figure S6: Scatter plot comparing the relative expression ratio for the 95 upregulated genes specifically expressed in the N0_1Y vs. N1_1Y comparison between wheat NILs under each N treatment; Figure S7: Scatter plot comparing the relative expression ratio for the 93 downregulated genes specifically expressed in the N0_1Y vs. N1_1Y comparison between wheat NILs under each N treatment; Figure S8: Comparison of GO classifications of DEGs between the two wheat NILs; Figure S9: Validation of 12 potential DEGs identified from transcriptome analysis by qRT-PCR; Figure S10: Gene expression correlation between the qRT-PCR and RNA-seq data. Table S1: Primer sequences used in this study; Table S2: Analysis of the DEGs in pairwise comparisons; Table S3: GO enrichment analysis of genes in pairwise comparisons; Table S4: Classification of N-Responsive Genes in 1Y revealed different responses to N-deficit conditions; Table S5: Category of N-Responsive Genes in 1W showed different responses to N deficiency.
