# Supplementary material for: Transcriptome Analysis Reveals Different Responsive Patterns to Nitrogen Deficiency in Two Wheat Near-Isogenic Lines Contrasting for Nitrogen Use Efficiency
Source: Biology (Basel). 2021 Nov 3;10(11):1126. doi: 10.3390/biology10111126 (PMC8614915; doi:10.3390/biology10111126)
Supplement: Supplementary file 1 [file biology-10-01126-s001.zip › Fig.pdf]

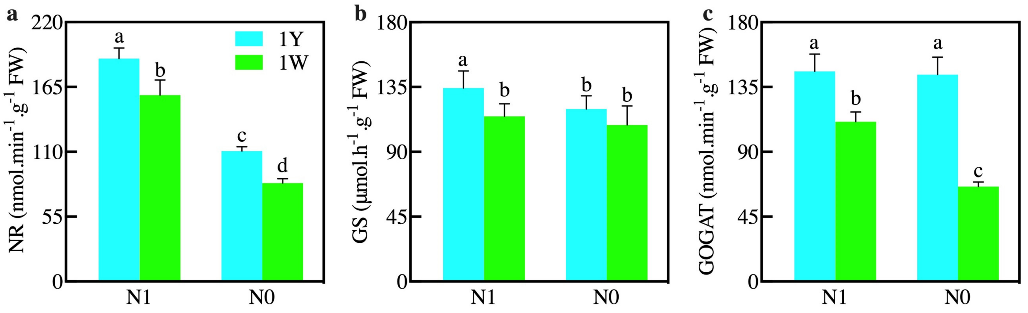

Figure S1 The activities of key enzymes related with N metabolism in the leaves of two wheat NILs under different N treatments. The error bars are the means ± SE (n = 3), and different letters represent significant difference ($p < 0.05$).

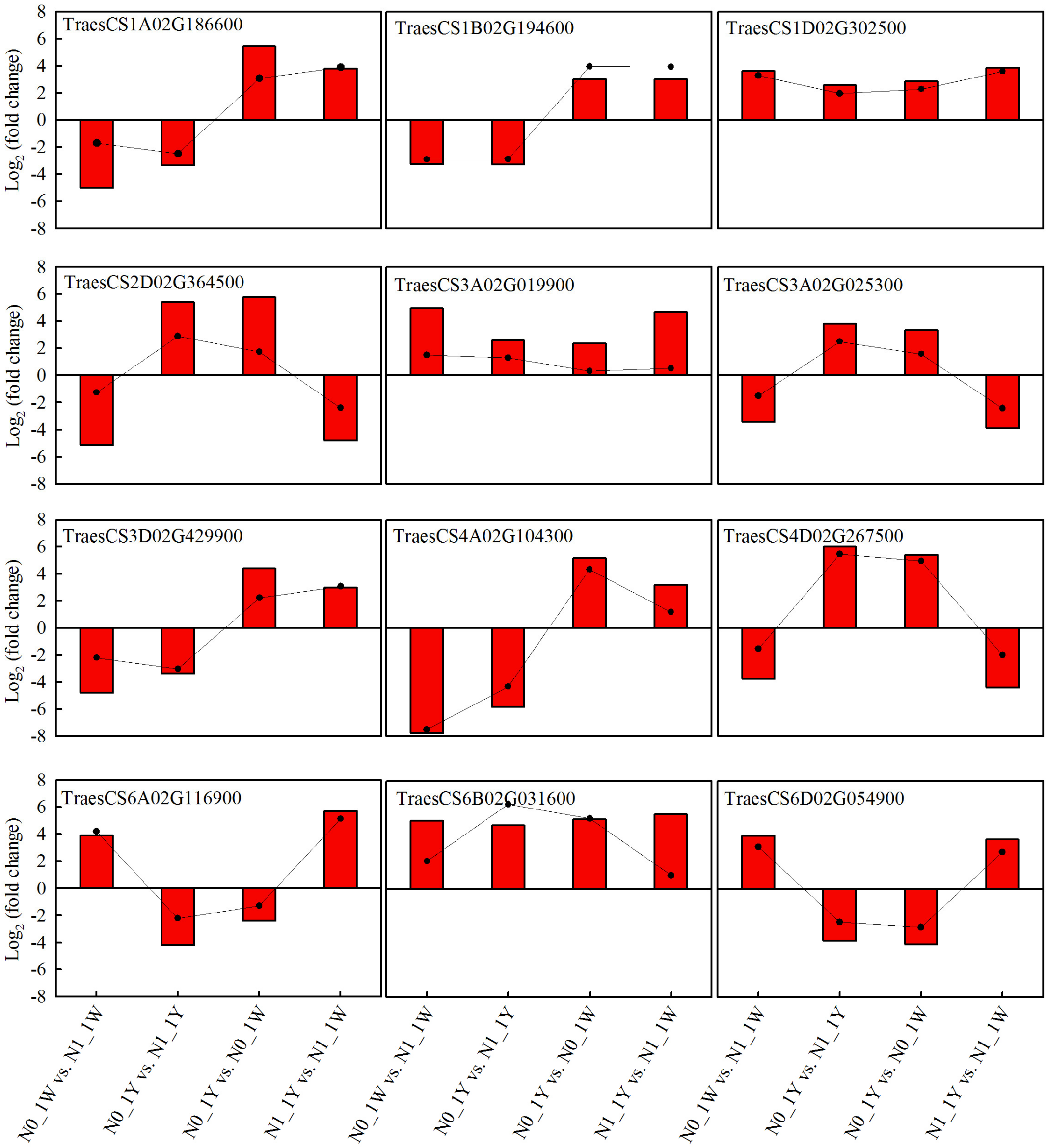

Figure S2 qRT-PCR validation of the RNA-seq data of the 12 randomly selected DEGs. The y-axis represents the gene relative expression levels. The red columns represent relative expression obtained by RNA-Seq, and the black solid lines represent relative expression obtained by qRT-PCR.

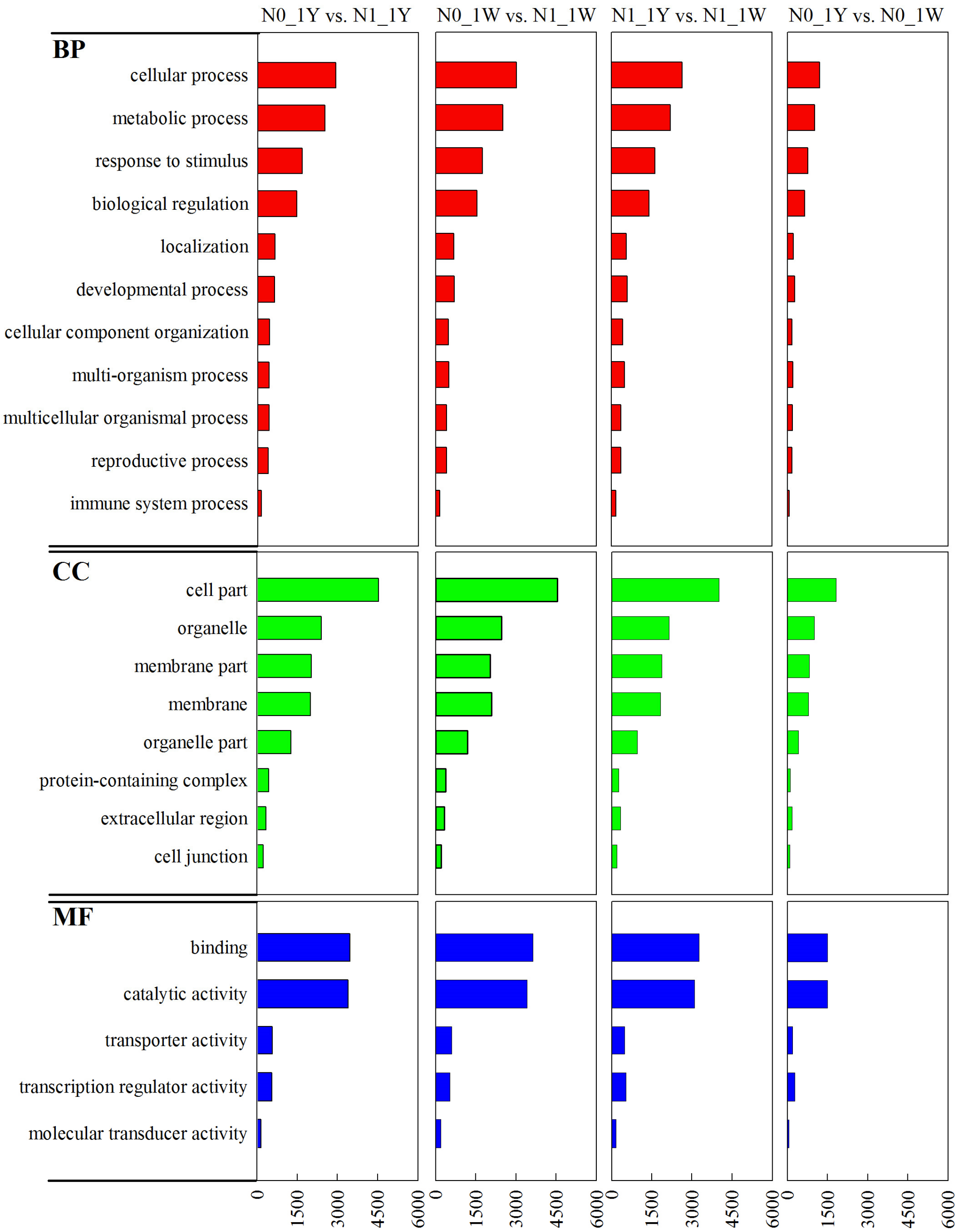

Figure S3. Distribution of DEGs among the GO terms in the three main categories. The *x*-axis shows the number of DEGs contained in each GO category, and the *y*-axis represents three main categories: biological process (BP), cellular component (CC), molecular function (MF).

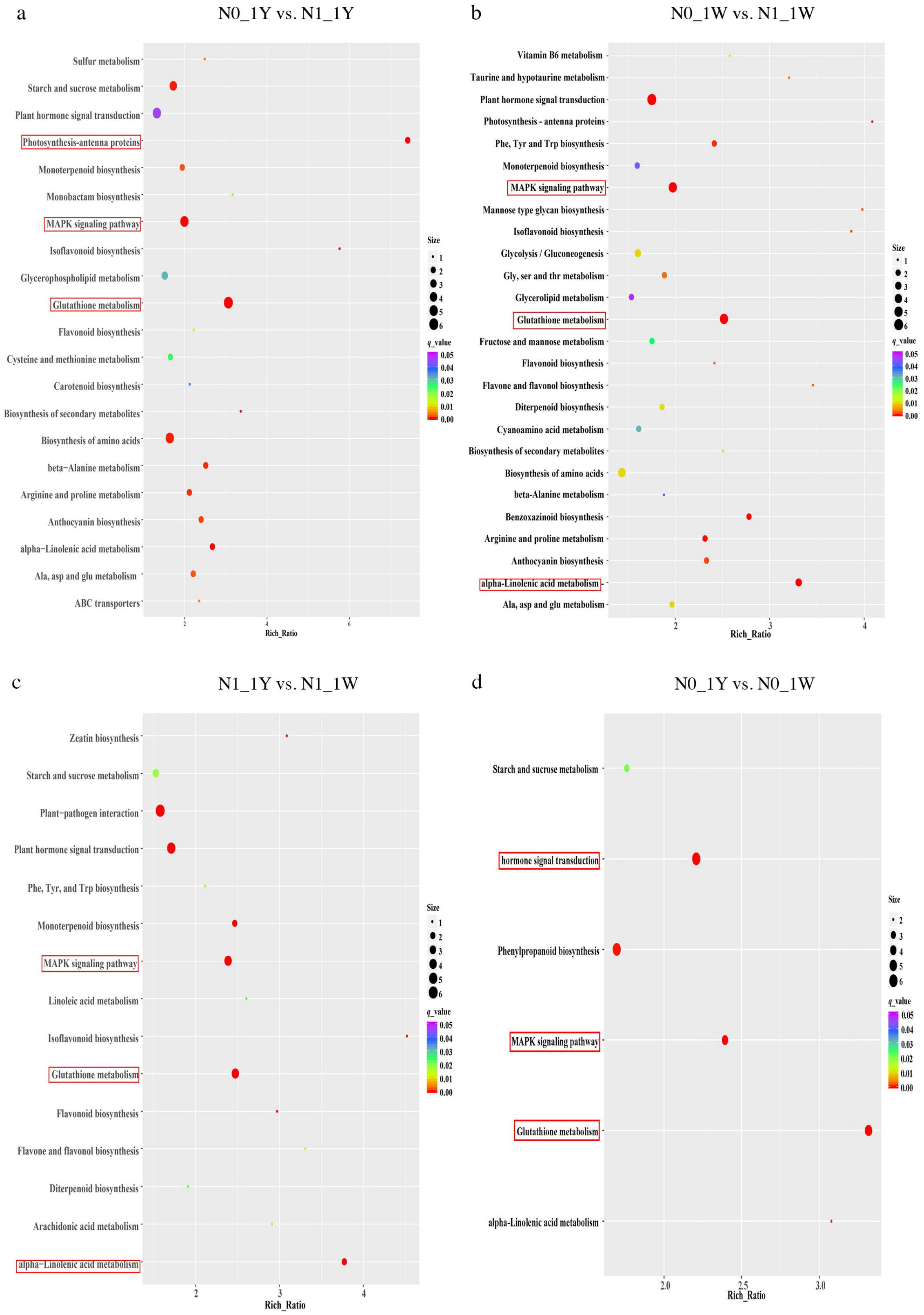

Figure S4. Analysis of KEGG enrichment in four pairwise groups. The *x*-axis indicates the Rich ratio, the *y*-axis represents the name of the KEGG pathway. The sizes of bubbles represent the number of DEGs of the pathway, and the colors of the bubbles represent the *q*-value. The color ratios and annotation are shown on the right side of the figure.

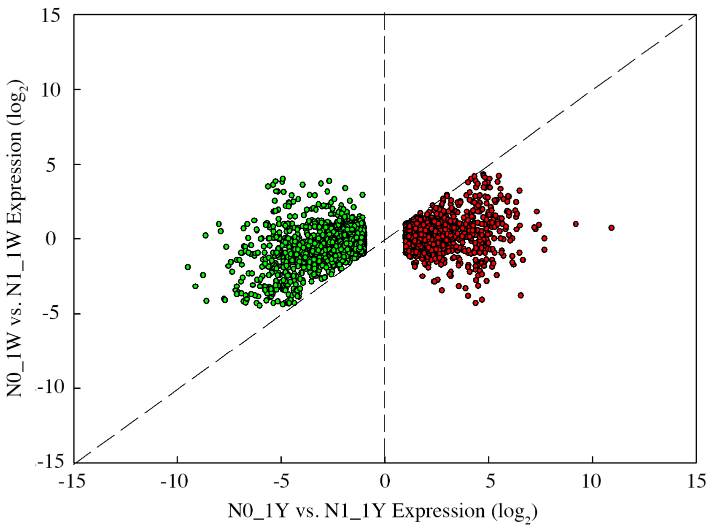

Figure S5. Scatter plot comparing the expression levels for the 4534 genes specifically expressed in the N0_1Y vs. N1_1Y comparison between N treatments in each wheat NIL. Each point represents an individual DEG, the red and green colors of the graph represent the genes that are upregulated or downregulated.

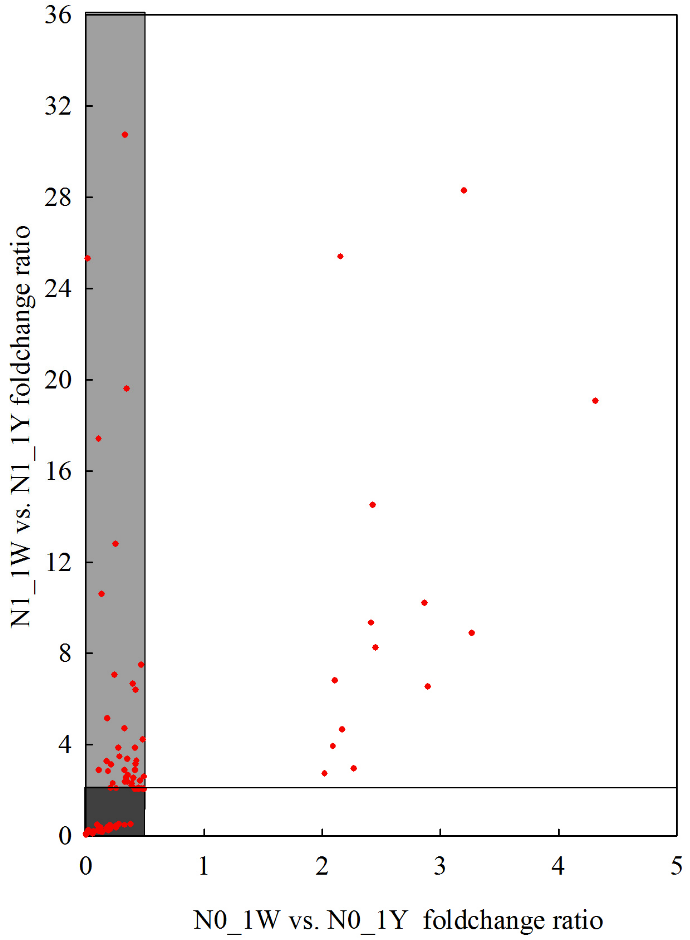

Figure S6. Scatter plot comparing the relative expression ratio for the 95 upregulated genes specifically expressed in the N0_1Y vs. N1_1Y comparison between wheat NILs under each N treatment. Each point represents an individual DEG, and the lighter section of the diagram shows the genes that are potentially frontloaded in expression.

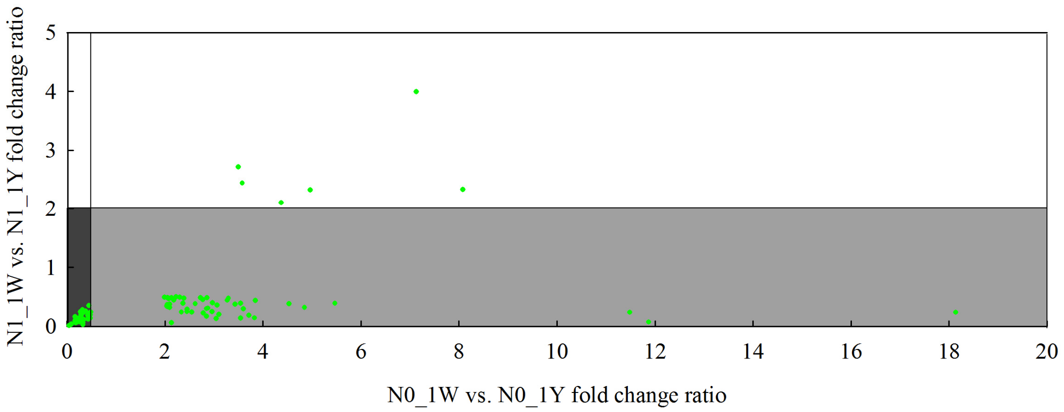

Figure S7. Scatter plot comparing the relative expression ratio for the 93 downregulated genes specifically expressed in the N0_1Y vs. N1_1Y comparison between wheat NILs under each N treatment. Each point represents an individual DEG, the lighter section of the diagram shows the genes that are potentially relatively upregulated in expression.

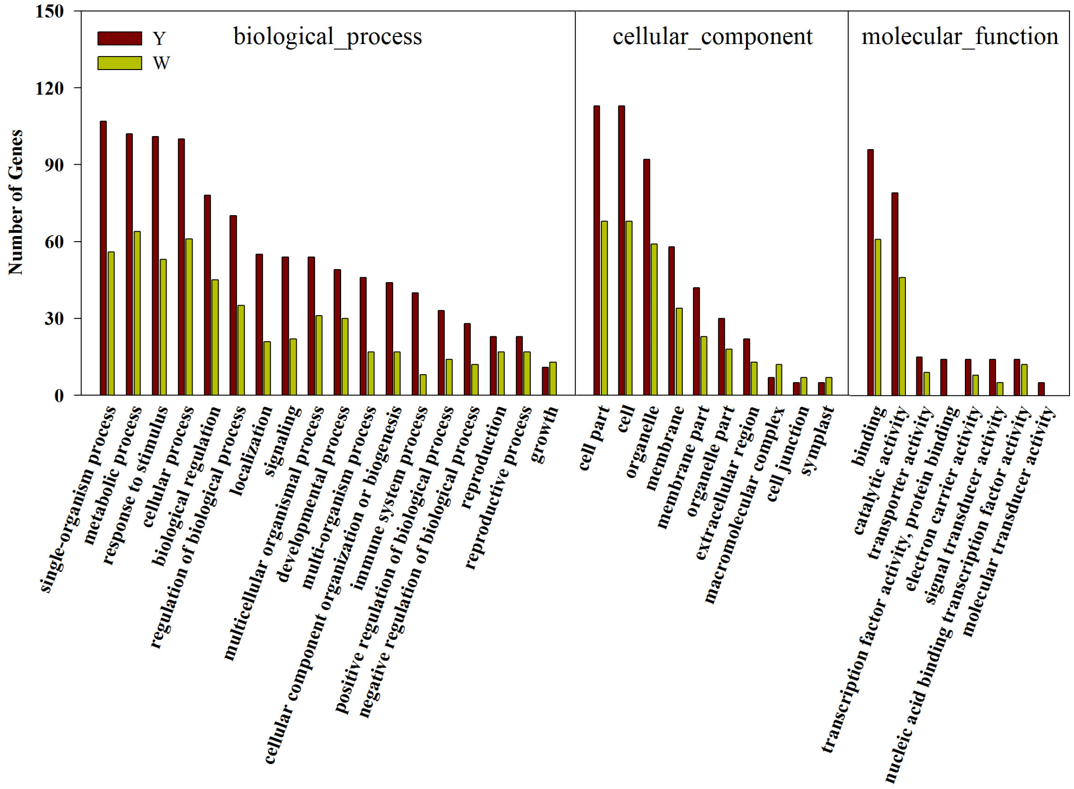

Figure S8. Comparison of GO classifications of DEGs between the two wheat NILs. The three main GO categories included biological process, cellular component, and molecular function.

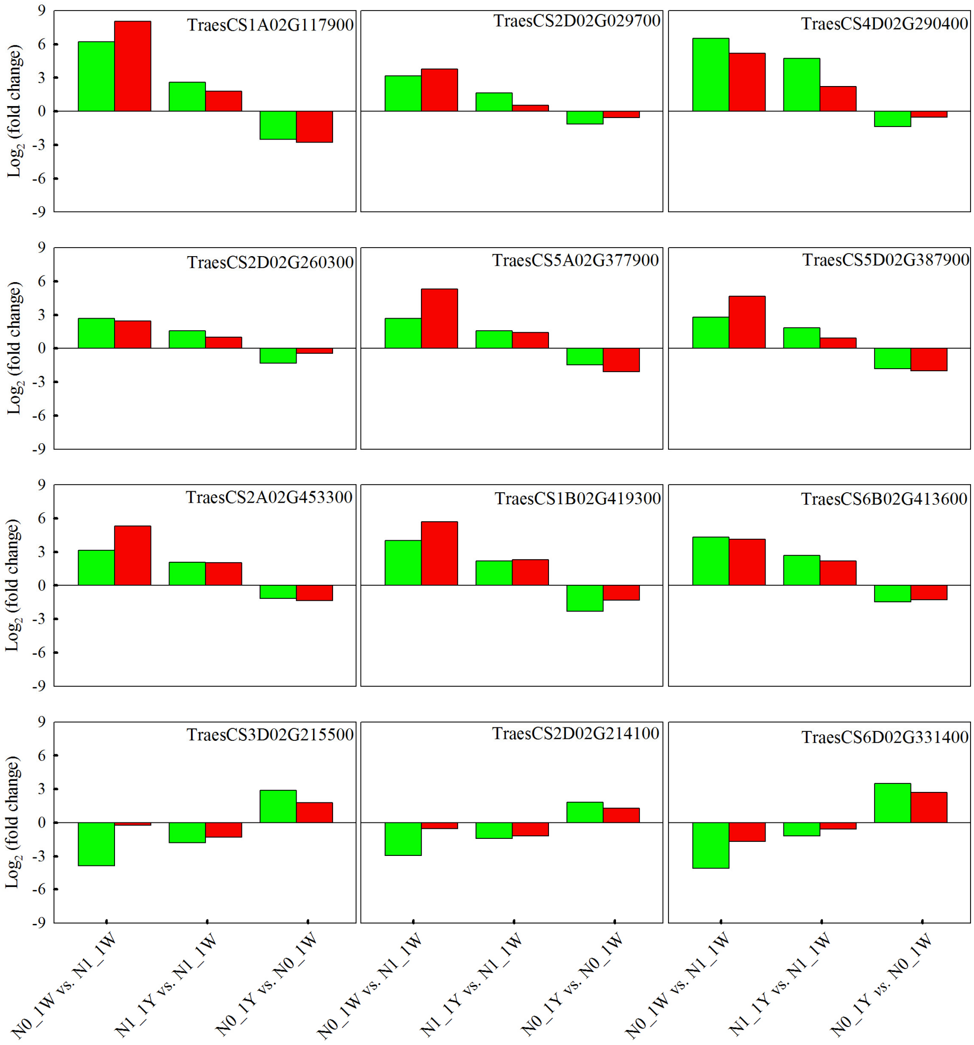

Figure S9. Validation of 12 potential DEGs identified from transcriptome analysis by qRT-PCR. The green column represents the gene relative expression levels in the RNA-Seq data, and the red column represents relative expression obtained by qRT-PCR.

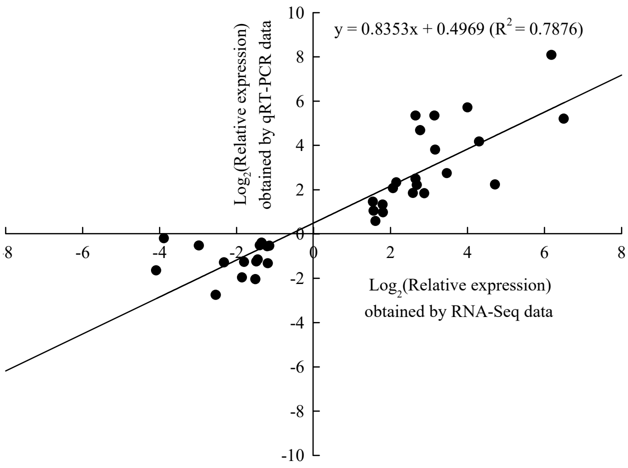

Figure S10. Gene expression correlation between the qRT-PCR and RNA-seq data. Each point represents a DEG, the x-axis stands for gene expression fold change in RNA-Seq, and the y-axis stands for gene expression fold change in qRT-PCR.
